# Improved Neurological Outcome in a Patient With Complicated Mitral Valve Endocarditis Treated With Daptomycin-Rifampin Regimen

**DOI:** 10.7759/cureus.23566

**Published:** 2022-03-28

**Authors:** Syed S Fatmi, Rafaela Basso, Ravitej Goteti

**Affiliations:** 1 Internal Medicine, Southeast Health Medical Center, Dothan, USA

**Keywords:** endocarditis, infectious endocarditis, cva, daptomycin, rifampin

## Abstract

Complicated infective endocarditis (IE) with symptomatic neurological involvement is associated with a poor prognosis. Vancomycin is the first-line antibiotic employed for the treatment of IE as general resistance of Methicillin-resistant *Staphylococcus aureus (*MRSA) to vancomycin is low and the antibiotic is well tolerated. In this case report, we describe a case of severely complicated MRSA endocarditis initially treated unsuccessfully with vancomycin. Our patient presented with severe encephalopathy with multiple septic infarctions noted on imaging. After treatment with a daptomycin-rifampin regimen, significant clinical improvement was noted. Based on the findings of this case report, what remains to be analyzed further with future studies is whether the daptomycin-rifampin regimen effect is independent of initial vancomycin-based treatment as most cases of IE are first treated with vancomycin and are only transitioned into daptomycin-rifampin regimen after treatment failure or persistent positive blood cultures, as is described in this case.

## Introduction

Endocarditis accounts for significant morbidity and mortality burden. According to some estimates, as much as 40% of overall mortality has been associated with complications of IE [1-3]. The most common causative pathogen of endocarditis is *Staphylococcus aureus*, which is seen in 23% to 68% of cases. Methicillin-resistant *S. aureus *(MRSA) carries a higher mortality burden compared to methicillin-susceptible *S*. *aureus* (MSSA) [4,5]. Complicated infective endocarditis is considered to be a relatively rare presentation and manifests itself as either embolization, mycotic aneurysm, or metastatic infection and can affect cardiovascular, nervous, renovascular, and other systems. Neurologic complications occur in 20% to 40% of patients with complicated endocarditis and are associated with a poor prognosis with increased mortality [1]. Neurological complications of IE can either be asymptomatic or present as encephalopathy, ischemic or hemorrhagic stroke [1,6]. These complications are primarily caused by vegetation emboli or rupture of a mycotic aneurysm, affecting mostly patients with mitral valve infections caused by *S. aureus*, *S. bovis*, Candida, or involvement of multiple valves, with one of the mentioned pathogens [7]. Complicated infective endocarditis with multiple septic emboli has been seen to be associated with adverse neurological outcomes in patients with symptomatic cerebrovascular accident (CVA) as compared to patients with non-symptomatic CVA or silent strokes, as neurological symptoms themselves are a representation of the severity of seeding of septic emboli. Vancomycin is usually considered the primary treatment modality, however, in this case report, we would like to highlight improved neurological outcomes in a patient treated with daptomycin-rifampin after treatment failure with the vancomycin regimen.

## Case presentation

A 55-year-old female presented with altered mental status, preceding fever associated with neck pain for 10 days. Before the commencement of fever, she was in her usual state of health. She was also found to have urine and fecal incontinence before presentation; however, the family denied any episodes of witnessed seizures or any associated sick contacts. Her past medical history was significant for depression and osteoarthritis. The patient was a former smoker, with a previous history of alcohol use until three years ago with no history of intravenous drug abuse. On physical examination, the patient was easily arousable, disoriented, unable to follow commands, and was moaning in pain. Vital signs showed blood pressure of 137/58 mmHg, pulse 102 beats per minute, respiratory rate of 16 breaths per minute, and temperature 103 °F. The patient had pain on passive flexion of the neck and no murmurs were noted on physical examination; however, the patient was found to have bilateral Janeway lesions and splinter hemorrhages. 

Labs and imaging

**Figure 1:**
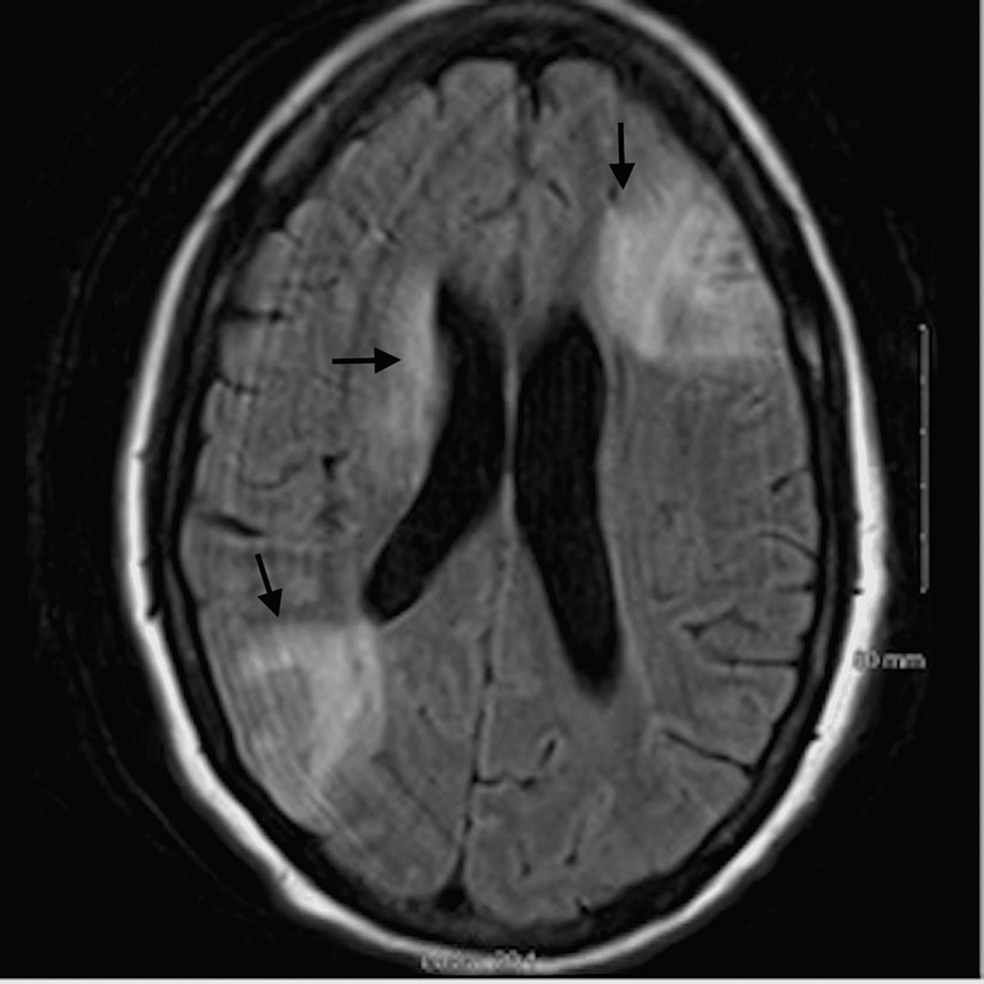
MRI Brain MRI image showing patchy acute infarctions with associated petechial hemorrhage and areas of leptomeningeal enhancement within the left frontal lobe and parietal lobe marked with arrows.

**Figure 2:**
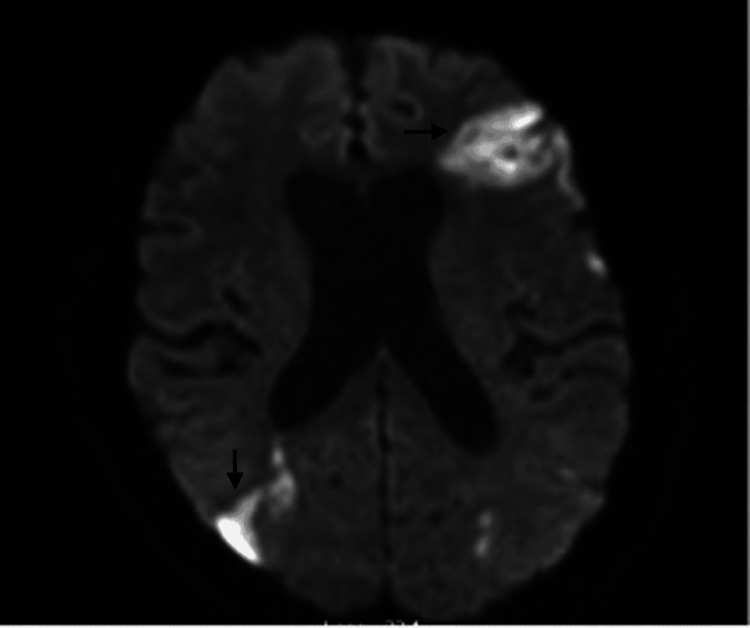
MRI Brain MRI image showing patchy acute infarctions within the left frontal lobe and right parietal lobe as indicated by arrows.

The initial laboratory workup showed troponin of 0.13 ng/mL, C-reactive protein (CRP) of 24.78 mg/L, hypomagnesemia, hypokalemia, negative meningitis, and encephalitis panel with positive blood culture for MRSA. Brain MRI showed patchy acute infarctions with associated petechial hemorrhage and areas of leptomeningeal enhancement within the left frontal lobe and bilateral parietal lobes concerning septic emboli (Figures 1, 2).

**Figure 3:**
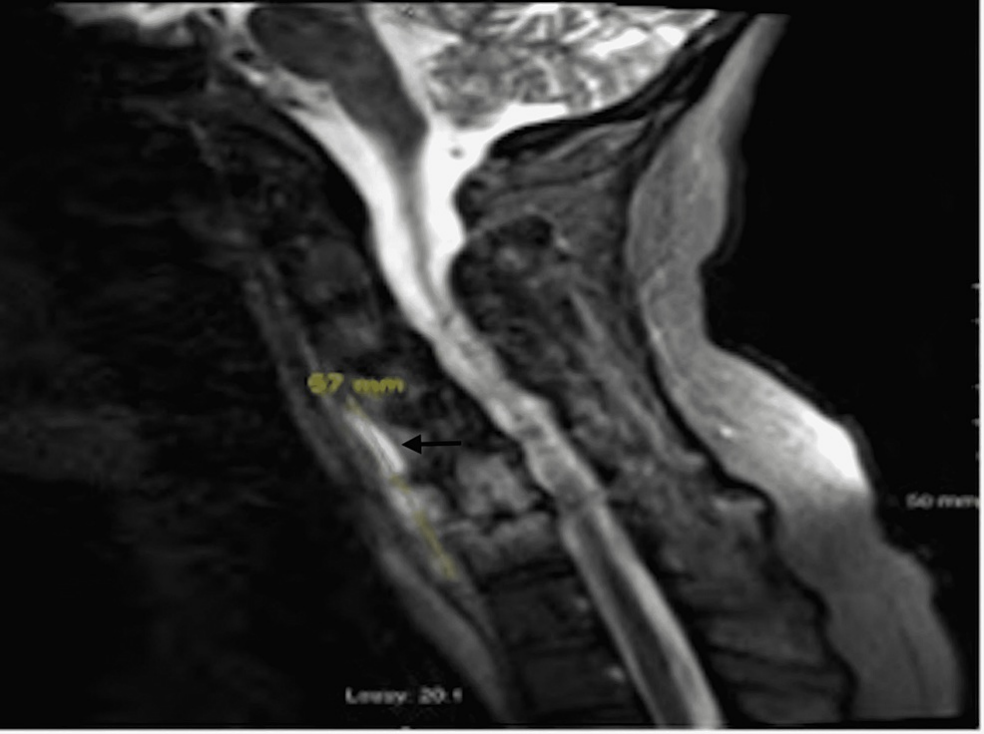
MRI Cervical Spine Image showing area of abnormal signal within the disc space and marrow at C5-C6 and C6-C7, marked by an arrow, concerning for discitis-osteomyelitis.

**Figure 4:**
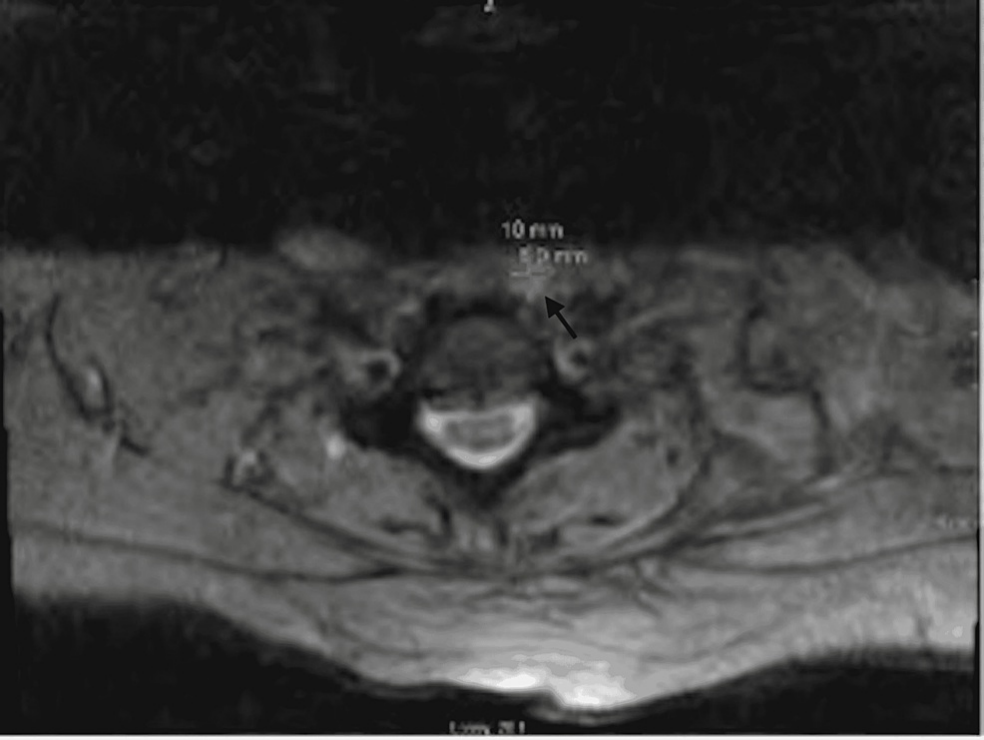
MRI Cervical Spine MRI image showing abnormal signal with prevertebral edema and fluid collection concerning for vertebral discitis-osteomyelitis with small prevertebral abscess, marked with an arrow.

MRI cervical spine showed abnormal signal within the disc space and marrow at C5-C6 and C6-C7, with prevertebral edema and fluid collection is concerning for vertebral discitis-osteomyelitis with small prevertebral abscess (Figures 3, 4). 

Course of medication

Although the patient did not have clear risk factors for IE, diagnosis of IE was confirmed after transesophageal echocardiogram (TEE) showed mitral valve vegetations, along with positive blood cultures and physical exam findings. The patient was initially treated with vancomycin. She continued to have persistent positive blood cultures for seven days after the initiation of vancomycin without any improvement in her clinical presentation. Vancomycin trough was closely monitored, and it was noted to be optimal and did not require dose adjustments. Subsequently, vancomycin was discontinued, as the patient continued to have positive cultures and the patient was started on daptomycin with rifampin. After 48 hours, the patient started showing an improvement in mentation. Through the course of the hospital stay, the patient showed a significant improvement in the overall clinical condition with a significant improvement in the neurological status including focal neurological deficits, mentation as well as functional status. With continued treatment with a daptomycin-rifampin regimen, the patient’s neurological status recovered to baseline with no residual focal neurological deficits. The patient was subsequently discharged after her neurological status had returned to baseline and at the time of discharge, the patient was continued on daptomycin and rifampin for a total of six weeks from the day of the first negative blood culture. The patient was followed up at the outpatient clinic two weeks after discharge and was found to be at her previous baseline functional status without any neurological or cognitive deficits. 

## Discussion

Although there is overwhelming evidence regarding the efficacy of the daptomycin-rifampin regimen over vancomycin [8,9], it needs to be taken into consideration that in numerous circumstances daptomycin is used after a patient fails to clear bacteremia on vancomycin and continues to have positive blood cultures. Based on these considerations, it still needs to be evaluated with future studies, whether the superiority of the daptomycin-rifampin regimen is independent of the supplemental effect of initial bacterial clearance with initial vancomycin treatment. Recommendations for utilization of daptomycin-rifampin as a first-line regimen with infective endocarditis continue to be vague, despite significant clinical evidence. Daptomycin-rifampin regimen has demonstrated significantly early bactericidal activity for MRSA as compared with vancomycin, where bactericidal activity is not achieved for up to 72 hours [10], which was also illustrated in our case with persistently positive blood cultures on vancomycin regimen. Based on previous studies, it is also quite evident that bacterial clearance is better achieved with daptomycin-rifampin and neurological outcomes were much more improved in patients who were treated with daptomycin-rifampin therapy, as compared to those treated with vancomycin alone. Daptomycin regimen also showed better long-term survival rates as well as decreased renal damage and acute kidney injury [11,12]. However, future studies are much required to assess and understand the mechanism for better neurological outcomes in the patients treated with the daptomycin-rifampin regimen with complicated infective endocarditis, while taking into consideration the complexity of the disease and associated complications such as patient's immune status and comorbidities. 

## Conclusions

Early and fast diagnosis and treatment of patients with IE prevent neurologic complications. Those who present with neurological complications have a poor prognosis with high mortality rates. Although it is quite evident, that daptomycin-rifampin therapy is efficacious in treating MRSA-related bacteremia and complicated IE, as illustrated by our case mentioned above, further studies are required to assess the independent efficacy of daptomycin with or without rifampin as a first line agent in treating complicated infective endocarditis.
